# Distribution of Peripheral Nerve Injuries in Patients with a History of Shoulder Trauma Referred to a Tertiary Care Electrodiagnostic Laboratory

**DOI:** 10.3390/diagnostics10110887

**Published:** 2020-10-30

**Authors:** Chul-Hyun Cho, Don-Kyu Kim, Du Hwan Kim

**Affiliations:** 1 Department of Orthopedic Surgery, Dongsan Medical Center, School of Medicine, Keimyung University, Daegu 42601, Korea; oscho5362@dsmc.or.kr; 2 Department of Physical Medicine and Rehabilitation, College of Medicine, Chung-Ang University, Seoul 06973, Korea; donkim21@gmail.com

**Keywords:** electrodiagnosis, shoulder dislocation, proximal humerus fracture, clavicle fracture, nerve injury, brachial plexus injury, axillary nerve

## Abstract

Peripheral nerve injury after shoulder trauma is an underestimated complication. The distribution of the affected nerves has been reported to be heterogeneous in previous studies. This study aimed to describe the distribution of peripheral nerve injuries in patients with a history of shoulder trauma who were referred to a tertiary care electrodiagnostic laboratory. A retrospective chart review was performed for all cases referred to a tertiary care electrodiagnostic laboratory between March 2012 and February 2020. The inclusion criteria were a history of shoulder trauma and electrodiagnostic evidence of nerve injury. Data on patient demographics, mechanism of injury, degree of weakness, clinical outcomes at the final follow-up, and electrodiagnostic results were retrieved from medical records. Fifty-six patients had peripheral nerve injuries after shoulder trauma. Overall, isolated axillary nerve injury was the most common. A brachial plexus lesion affecting the supraclavicular branches (pan-brachial plexus and upper trunk brachial plexus lesions) was the second most common injury. In cases of shoulder dislocation and proximal humerus fracture, isolated axillary nerve injury was the most common. Among acromioclavicular joint injuries and clavicular fractures, lower trunk brachial plexus injuries and ulnar neuropathy were more common than axillary nerve or upper trunk brachial plexus injuries. Patients with isolated axillary nerve lesions showed a relatively good recovery; those with pan-brachial plexus injuries showed a poor recovery. Our study demonstrated the distribution of peripheral nerve injuries remote from displaced bony structures. Mechanisms other than direct compression by displaced bony structures might be involved in nerve injuries associated with shoulder trauma. Electrodiagnostic tests are useful for determining the extent of nerve damage after shoulder trauma.

## 1. Introduction

The brachial plexus and its branches originate from the cervical spine, pass through the shoulder girdle, and run through the entire upper extremity [1]. The glenohumeral joint of the shoulder is a shallow ball and socket joint and is the joint with the highest mobility in the human body [2]. Shoulder trauma can cause various types of injury, such as glenohumeral joint dislocation, acromioclavicular joint injury, humeral fracture, clavicular fracture, and soft tissue injury, including rotator cuff tear [3,4]. Due to the anatomic proximity of the shoulder joint and brachial plexus and its branches, these injuries can affect neural structures around the shoulder joint. The incidence of nerve injury following shoulder dislocation or humeral neck fracture has been found to be 35–65%, but the distribution of affected nerves has been reported to be heterogeneous depending on the reported study [5,6,7,8,9]. Hems and Mahmood reported that shoulder dislocation was associated with injuries to the terminal branches of the infraclavicular brachial plexus, but Kosiyatrakul et al. demonstrated that two-thirds of cases had total brachial plexus injuries, suggesting supraclavicular brachial plexus lesions [10,11].

It is not easy to determine whether patients have peripheral nerve injuries immediately after shoulder trauma because of the use of immobilization in treatment [6]. Subsequent dysfunction of the shoulder following peripheral nerve injury is often mistaken for immobilization-related contracture. It is important to identify the presence and severity of peripheral nerve injuries because rehabilitative intervention at the right time may be crucial depending on the severity of the injury, and the physician can inform the patient of the prognosis of peripheral nerve injuries.

This study aimed to describe the distribution of peripheral nerve injuries in patients with a history of shoulder trauma referred to a tertiary care electrodiagnostic laboratory and to review the literature concerning nerve injuries following shoulder trauma.

## 2. Materials and Methods

A retrospective chart review was performed for all cases referred to a tertiary care electrodiagnostic laboratory between March 2012 and February 2020. Inclusion criteria were as follows: (1) a history of shoulder trauma, (2) weakness of the upper limb on physical examination, (3) appropriate electrodiagnostic work-up, (4) electrodiagnostic evidence of nerve injury, and (5) follow-up for a minimum of 1 year. Shoulder trauma included proximal humeral fracture, glenohumeral dislocation with/without accompanying injuries such as fracture of the greater tubercle of the humerus, bony Bankart lesion, acromioclavicular joint injury, clavicular fracture, scapular fracture, high-energy blunt trauma, and stretching injury. Exclusion criteria were as follows: (1) previous weakness of the upper limb; (2) concomitant cervical spinal cord injury or weakness due to trauma-related disc herniation proven by magnetic resonance imaging; (3) non-traumatic cause of shoulder weakness such as neuralgic amyotrophy or cervical radiculopathy due to foraminal stenosis or non-traumatic disc herniation; and (4) incomplete electrodiagnostic work-up. The demographic data of the patients, mechanism of injury, degree of weakness, clinical outcomes at final follow-up, and electrodiagnostic results were retrieved from medical records.

On presentation to the electrodiagnostic laboratory, focused history taking regarding the mechanism of trauma was performed, and all patients underwent neurologic and musculoskeletal examination. Active and passive range of motion, motor, and sensory function in the upper extremities were routinely examined. Special attention was paid to the strength of the shoulder prime mover, as well as distal upper extremities. A low-energy fracture was defined as that which occurred as a result of falling from standing height or less, while a high-energy fracture was defined as any other type of trauma (e.g., falling from a height greater than standing height, motor vehicle accident).

Electrodiagnostic tests were usually performed during conservative treatment or postoperatively in cases involving significant neurologic deficits based on the judgment of the orthopedic surgeon. Appropriate electrodiagnostic work-up included motor nerve conduction studies (NCS) on the median, ulnar, radial, suprascapular, axillary, and musculocutaneous nerves; sensory NCS on the median, ulnar, superficial radial, lateral antebrachial cutaneous, and medial antebrachial cutaneous nerves; and needle electromyography (EMG) of the muscles of the upper limb and cervical multifidus. Based on the electrodiagnostic work-up, the pattern of peripheral nerve injury was classified as root lesion, brachial plexopathy, isolated mononeuropathy, or multiple nerve injury.

Patients’ clinical outcomes were classified as no impairment (subjectively satisfied, no problems in activities of daily living (ADLs)), mild impairment (subjectively not satisfied, no problems with ADLs), and impairment (subjectively not satisfied, problems with ADLs) [2].

This study was approved by the Institutional Review Board of Dongsan Medical Center (No. 2020-07-024, approved on 13 July 2020).

## 3. Results

The type of shoulder joint injury could be divided as follows: (1) shoulder dislocation, (2) proximal humerus fracture, (3) acromioclavicular injury including clavicular fracture, and (4) miscellaneous.

### 3.1. Detailed Descriptions of Peripheral Nerve Injury after Shoulder Dislocation (n = 17)

There were 17 patients with nerve injury associated with shoulder dislocation, including eight men and nine women with a mean age of 54 years (16–75 years). The mean time between injury and electrodiagnostic work-up was 2.9 months. Among the 17 shoulder dislocations, 11 resulted from low-energy trauma, with the mechanism of injury being a fall from a standing height in six patients, a fall from a height in two patients, a road traffic accident in one patient, and a sports accident in two patients. Six patients experienced high-energy trauma: a fall from tree height in two patients, a motor vehicle accident in two patients, and a mountain biking accident in two patients. A rotator cuff tear or displaced fracture of the greater tubercle was present in seven patients (41.2%).

The involved nerve, electrodiagnostic results, and recovery details are shown in Table 1. Seven patients had an isolated axillary nerve injury, three patients had a pan-brachial plexus lesion, two patients had an isolated median nerve injury, one patient had a C5-6 root injury, and four patients had a multiple nerve injury. All four patients with a multiple nerve injury had axillary nerve lesions combined with a musculocutaneous nerve injury (*n* = 2), a suprascapular nerve injury (*n* = 1), or a C5-6 root injury (*n* = 1). Overall, axillary nerve injury was the most common nerve injury associated with shoulder dislocation. Most isolated axillary nerve lesions resulted from low-energy trauma, but pan-brachial plexus lesions were associated with high-energy trauma.

Complete follow-up was performed to ensure that all patients fully recovered from the injury or were monitored for at least one year. All three patients with pan-brachial plexus had problems with ADLs. The seven patients with isolated axillary nerve lesions showed relatively good recoveries, with no impairment in three patients, mild impairment in three, and impairment in one.

### 3.2. Detailed Descriptions of Peripheral Nerve Injury after Proximal Humerus Fracture

There were 19 patients with nerve injury associated with proximal humerus fracture excluding fracture of the greater tubercle. All 19 patients had displaced fractures, with glenohumeral joint dislocation in three. There were 10 men and 9 women with a mean age of 54 years (18 to 92 years). The mean time between injury and electrodiagnostic work-up was 2.9 months. Among the 19 proximal humerus fractures, seven resulted from low-energy trauma, with the mechanism of injury being a fall from a standing height in all patients. Twelve patients experienced high-energy trauma, with a fall from tree height in five, motor vehicle accident in three, and a mountain biking accident in two, a pedestrian accident in one, and a motorcycle accident in one.

The involved nerve, electrodiagnostic results, and recovery details are shown in Table 2. Nine patients had an isolated axillary nerve injury, two patients had pan-brachial plexus lesions, two patients had isolated ulnar nerve injury, and six patients had multiple nerve injury. The axillary nerve was affected in all six patients with multiple nerve injury, and was accompanied by musculocutaneous nerve injury in four patients, median nerve injury in one, and ulnar nerve injury in one. The most common nerve injury associated with proximal humerus fracture was the axillary nerve.

All patients were followed-up for at least one year. Incomplete recovery was observed in two patients with pan-brachial plexus injuries. Nine patients with an isolated axillary nerve lesion showed a relatively good recovery, with no impairment in four patients and mild impairment in five. Six patients with multiple nerve injuries also had a relatively good recovery, with no impairment in one patient, mild impairment in four, and impairment in one.

### 3.3. Detailed Description of Peripheral Nerve Injury after Acromioclavicular Injury

There were 12 patients with nerve injury associated with acromioclavicular injury. Eight patients had a clavicular fracture, and four patients had an acromion fracture or acromioclavicular dislocation. There were eight men and four women with a mean age of 47 years (18 to 84 years). The mean time between injury and electrodiagnostic work-up was 2.6 months. All patients experienced high-energy trauma, with a fall from over standing height in three, motor vehicle accident in six, and motorcycle accident in three.

The involved nerve, electrodiagnostic results, and recovery details are shown in Table 3. Five patients had lower trunk brachial plexus lesions, two had mainly upper trunk brachial plexus lesions, two had isolated ulnar nerve lesions, two had isolated radial nerve lesions, and one had an axillary nerve lesion. Most patients had a relatively good recovery, with no impairment in six patients, mild impairment in five, and impairment in one.

### 3.4. Miscellaneous

Eight patients had nerve injuries associated with various traumatic conditions. Three patients had a nerve injury associated with a traumatic rotator cuff tear, three had a nerve injury associated with scapular body fracture, and two had a nerve injury associated with an open wound. The mean time between injury and electrodiagnostic work-up was 5.2 months.

The involved nerve, electrodiagnostic results, and recovery details are shown in Table 4. Of the three patients with traumatic rotator cuff tears, one had a C5-6 root injury, one had an axillary nerve injury, and one had an upper trunk brachial plexus injury. All three patients with scapular body fractures had upper trunk brachial plexus injuries.

Table 5 summarizes the patterns and prognosis of nerve injury based on the electrodiagnosis in patients with shoulder trauma. The most common nerve injury associated with shoulder trauma was isolated axillary nerve injury, followed by axillary nerve injury combined with a musculocutaneous nerve injury, pan-brachial plexus injury, and upper trunk brachial plexus injury.

## 4. Discussion

From the perspective of an electrodiagnostic specialist, it is important to evaluate which nerves were affected and how severely the nerves were damaged in patients with a history of shoulder trauma and subsequent neurologic deficit. For example, if the patient has a weakness in shoulder abduction and external rotation, it is important to determine whether the neurologic deficit is caused by an isolated axillary nerve injury, an axillary nerve combined with a suprascapular nerve injury, or an upper trunk injury of the brachial plexus and to judge whether the nerve injury is incomplete or complete. The decision of the electrodiagnostic specialist would guide future examinations and the necessity of subsequent surgical treatment, such as explorative neurolysis, nerve resection and grafting, or tendon transfer [12]. As patients with shoulder trauma tend to be immobilized as an acute phase treatment, it is difficult to accurately evaluate muscle weakness around the shoulder. Electrodiagnostic work-up can reveal the presence of nerve injury after shoulder trauma. This present study described the distribution of nerve damage according to the type of shoulder trauma and reviewed the literature related to nerve lesions after shoulder trauma.

Previous cross-sectional studies have demonstrated various types of peripheral nerve injuries after shoulder trauma (Table 6) [4,6,8,9,10,13,14,15]. In this study, the distribution of nerve injuries was similar to those in previous studies: the axillary nerve is the most commonly affected nerve as a result of shoulder dislocation and proximal humerus fracture. However, contrary to the results of previous studies, brachial plexus lesions affecting the supraclavicular branches (pan-brachial plexus and upper trunk brachial plexus lesions) were the second most common type of injury. Injury of the lower trunk of the brachial plexus and ulnar neuropathy were more common than axillary nerve injury or upper trunk brachial plexus injury in patients with acromioclavicular joint injury or clavicular fracture.

Anterior dislocation of the shoulder results from a combination of abduction, extension, external rotation, and a posteriorly directed force applied to the arm, levering the humeral head out of the glenoid [10]. The axillary nerve is vulnerable to being stretched over the humeral head under tension if the shoulder is over-abducted and the humeral head is forced downwards. Birch reported that “injury to the infraclavicular plexus associated with anterior dislocation of the shoulder is produced by intrusion of the head of the humerus into the axilla, beneath the cords and terminal branches of the plexus” [16]. In displaced proximal fractures, the end of the humeral shaft directly compresses on the neurovascular bundle in the axilla, leading to nerve injuries [6,10]. If the displaced bony fragment is not reduced at the right time, ongoing pressure on the neurovascular bundle may cause permanent paralysis.

There is no consensus regarding the frequency of associated nerve lesions following shoulder dislocation and proximal humerus fracture. Most reviews on the neurological complications of shoulder dislocation focus on infraclavicular postganglionic injuries, especially isolated nerve injuries [4,17,18]. There is no doubt that isolated axillary nerve injury is the most common neurological complication in patients with shoulder dislocation and proximal humerus fracture. Robinson et al. described that isolated ulnar and median nerve injuries were the next two most common lesions [4]. Hems and Mahmood also reported that ulnar nerve injuries were the second most common injury, and the outcome of ulnar nerve injury at the level of the shoulder was poor [10]. However, several studies have demonstrated that the suprascapular nerve or radial nerve were more commonly injured than the ulnar nerve [6,9]. These heterogeneous results suggest that various forces are transmitted to shoulder structures at the time of shoulder trauma, and mechanisms other than direct compression might be involved in nerve injury. Brachial plexus injury after shoulder dislocation or proximal humerus fracture is less common than isolated axillary nerve injury. Owing to the rarity of surveys based on meticulous electrodiagnostic examinations, the true incidence of brachial plexus injury other than isolated nerve injuries after shoulder dislocation or proximal humerus fracture is rarely reported in the literature. However, brachial plexus lesions affecting the supraclavicular branches (pan-brachial plexus and upper trunk brachial plexus lesions) were the second most common type of injuries in our study.

Considering the proximity between deranged bony structures and nerves, brachial plexus lesions following shoulder dislocation and proximal humerus fracture have been explained by three mechanisms: (1) traction on neural structures from the displaced humeral head or shaft, (2) mechanical compression of the brachial plexus by bony structures, and (3) compression by hematoma or fibrosis [13,19]. Beyond these mechanisms, we postulate the possibility that significant forces applied to or across the head and shoulder girdle at the time of trauma could lead to stretching or traction injury of the brachial plexus, irrespective of the displacement of the humeral head or shaft. Our study demonstrated that 8 (22.2%) of 36 patients with shoulder dislocation and proximal humerus fracture had injuries of the terminal branches of the supraclavicular brachial plexus, remote from the shoulder pathology. It seems that initial high-energy trauma might contribute to injury to the supraclavicular brachial plexus. The contribution of the energy of the initial trauma to nerve injury seems to be controversial. For example, the largest population sample study examining traumatic anterior glenohumeral dislocation demonstrated that neurologic deficits associated with shoulder dislocation were greater in patients who had experienced low-energy trauma [4]. In contrast, Visser et al. reported that the incidence of nerve injury associated with proximal humerus fracture (68%) is greater than that of shoulder dislocation alone (50%), suggesting a relationship between the energy of the initial trauma and nerve injury [9]. Young patients tend to be exposed to higher-energy trauma than older patients, but they have a wider tolerance to stretching injury exceeding the tissue elastic limit and a higher regenerative capacity.

Although clavicle fractures are common, the incidence of nerve injury associated with clavicle fracture has rarely been reported [20]. Brachial plexus injury after clavicle fracture is usually due to hypertrophic callus formation; thus, it is a late complication of clavicle fracture. Brachial plexus injury within 1 month after clavicular fracture is extremely rare. Della Santa et al. reported that the incidence of brachial plexus lesions due to clavicle fractures represented approximately 1% of lesions during a 20-year period [21]. Most cases with brachial plexus injury after clavicle fracture have been reported as case reports [20,22,23]. Exceptionally, Hill et al. reported that 15 of 52 patients with severely displaced fractures of the clavicle had some evidence of thoracic outlet syndrome, but the authors did not give any information regarding electrodiagnostic results [24]. Previous studies demonstrated that the medial and posterior cords were most frequently involved in clavicle fracture-related brachial plexus injury [21,25]. In this study, the distribution of patterns of nerve injury following acromioclavicular injury, including clavicle fracture, varied, suggesting that various external forces influenced nerve injuries besides compression by bony fragments or hypertrophic callus formation. To identify these concomitant nerve injuries remote from the clavicle fracture may predict the residual loss of limb function that would persist after corrective surgery.

This study has several limitations. First, this study was a retrospective design. Second, patients referred to the electrodiagnostic laboratory were enrolled. Thus, this study could not survey the true incidence of peripheral nerve injury after shoulder trauma. Third, follow-up electrodiagnosis was not performed. Fourth, the interval from onset to electrodiagnosis was somewhat heterogeneous. Fifth, various confounding factors did not enable the analysis of the correlation between recovery and several variables. However, it is meaningful to describe the distribution of peripheral nerve injuries according to the type of shoulder joint injury.

## 5. Conclusions

In conclusion, various patterns of peripheral nerve injury after shoulder trauma were noted, although the most common nerve injury was isolated axillary nerve injury. Brachial plexus lesions affecting the supraclavicular branches (pan-brachial plexus and upper trunk brachial plexus lesions) were also common. Patients with isolated axillary nerve lesions showed a relatively good recovery. Patients with pan-brachial plexus injury showed a poor recovery. Electrodiagnostic tests are useful in determining the extent of nerve damage after shoulder trauma.

## Figures and Tables

**Table 1:** Detailed Description of the Peripheral Nerve Injuries in 17 Patients with Shoulder Dislocation.

No	Sex/Age	Energy	Interval to EDX (Months)	Degree of Weakness at Onset	Accompanying Injury	Motor NCS Abnormality	Denervated Muscles on Needle EMG	Affected Nerves	Recovery
1	F/72	Low	12	Incomplete	RCT	AN	D	AN	Mild impairment
2	F/65	High	2	Complete	GTF	AN, MCN	Multifidus, BB, D, SSP	C5-6 root	Mild impairment
3	F/72	High	1.5	Complete	GTF	RN, UN, SSN	D, BB, BR, TB, FCR, FCU APB, FDI	pan-brachial plexus	Impairment
4	M/16	Low	1.5	Incomplete	–	None	D	AN	No impairment
5	F/74	Low	1	Incomplete	RCT	AN	D	AN	Impairment
6	M/64	Low	3	Incomplete	–	AN, MCN	D, BB	AN, MCN	Impairment
7	M/56	Low	0.5	Incomplete	–	AN, MCN	D, BB	AN, MCN	Mild impairment
8	F/60	Low	3	Incomplete	–	AN	D	AN	Mild impairment
9	M/48	High	1	Incomplete	–	AN	Multifidus, BB, D, SSP	AN, C5-6 root	No impairment
10	M/65	High	0.5	Incomplete	GTF	RN, UN, AN	D, TB, FCR, FCU APB, FDI	pan-brachial plexus	Mild impairment
11	F/75	High	3	Incomplete	–	AN	D	AN	Mild impairment
12	F/69	High	1	Complete	RCT	MN, UN, RN, MCN	D, BB, BR, TB, FCR, FCU APB, FDI	pan-brachial plexus	Impairment
13	M/43	Low	12	Incomplete	–	AN, SSN	D, SSP	AN, SSN	Mild impairment
14	F/75	Low	4	Complete	HF	MN	FCR, FDS, PQ, APB	MN	Impairment
15	M/43	Low	1	Incomplete	–	MN (PQ recording)	PQ, FPL	AIN	No impairment
16	M/29	Low	1	Incomplete	–	None	D	AN	No impairment
17	M/36	Low	1	Incomplete	–	AN	D	AN	No impairment

EDX, electrodiagnosis; NCS, nerve conduction study; EMG, electromyography; RCT, rotator cuff tear; GTF, greater tubercle fracture; HF, humerus fracture; AN, axillary nerve; MCN, musculocutaneous nerve; RN, radial nerve; SSN, suprascapular nerve; MN, median nerve; AIN, anterior interosseous nerve; SSP, supraspinatus; D, deltoid; BB, biceps brachii; BR, brachioradialis; TB, triceps brachii; FCR, flexor carpi radialis; FCU, flexor carpi ulnaris; APB, abductor pollicis brevis; FDI, first dorsal interosseous; PQ, pronator quadratus; FPL, flexor pollicis longus.

**Table 2:** Detailed Description of the Peripheral Nerve Injuries in 19 Patients with Proximal Humerus Fracture.

No	Sex/Age	Energy	Interval to EDX (Months)	Degree of Weakness at Onset	Motor NCS Abnormality	Denervated Muscles on Needle EMG	Affected Nerve	Recovery
1	M/57	High	10	Incomplete	UN, AN	D, FCU, FDI	AN, UN	Mild impairment
2	M/18	High	4	Complete	MN, UN, RN, AN, MCN	D, BB, SSP, BR, TB, FCR, FCU APB, FDI	Pan-brachial plexus	Impairment
3	M/30	Low	2	Incomplete	AN	D	AN	No impairment
4	F/62	Low	1.5	Incomplete	AN, MCN	D, BB	AN, MCN	Mild impairment
5	M/38	High	3	Complete	MN, AN	D, FCR, APB	AN, MN	Impairment
6	M/78	Low	0.7	Incomplete	AN, MCN	D, BB	AN, MCN	Impairment
7	M/23	High	1	Incomplete	AN, MCN	D, BB	AN, MCN	No impairment
8	F/92	Low	0.5	Complete	MN, UN, RN,	D, BB, TB, FCU, FDI	Pan-brachial plexus	Impairment
9	F/54	High	2	Incomplete	AN	D	AN	Mild impairment
10	M/76	Low	2	Incomplete	UN	FCU, FDI	UN	Mild impairment
11	F/43	High	1	Incomplete	AN, MCN	D, BB	AN, MCN	Mild impairment
12	M/38	High	1	Incomplete	AN	D	AN	No impairment
13	F/81	High	12	Incomplete	AN	D	AN	No impairment
14	M/53	Low	6	Incomplete	AN	D	AN	Mild impairment
15	M/64	High	2	Incomplete	None	D	AN	No impairment
16	F/38	Low	1	Incomplete	AN	D	AN	Mild impairment
17	F/57	High	1	Complete	UN	FCU, FDI	UN	Impairment
18	F/73	High	2	Incomplete	AN	D	AN	Mild impairment
19	F/62	High	2	Incomplete	AN	D	AN	Mild impairment

EDX, electrodiagnosis; NCS, nerve conduction study; EMG, electromyography; AN, axillary nerve; MCN, musculocutaneous nerve; RN, radial nerve; MN, median nerve; AIN, anterior interosseous nerve; UN, ulnar nerve; SSP, supraspinatus; D, deltoid; BB, biceps brachii; BR, brachioradialis; TB, triceps brachii; FCR, flexor carpi radialis; FCU, flexor carpi ulnaris; APB, abductor pollicis brevis; FDI, first dorsal interosseous.

**Table 3:** Detailed Description of the Peripheral Nerve Injuries in 12 Patients with Acromioclavicular Injuries.

No	Sex/age	Energy	Injury Area	Interval to EDX (Months)	Degree of Weakness at Onset	Motor NCS Abnormality	Denervated Muscles on Needle EMG	Affected Nerve	Recovery
1	F/52	High	Clavicle Fx	2	Incomplete	UN	FCU, FDI	UN	Mild impairment
2	M/47	High	Clavicle Fx	3	Complete	AN	D	AN	Impairment
3	M/18	High	Clavicle Fx	1	Incomplete	RN	TB, EDC	RN	No impairment
4	M/45	High	Clavicle Fx	2	Incomplete	None	TB, EDC	RN	No impairment
5	F/47	High	Acromioclavicular joint dislocation	5	Incomplete	None	FDI, APB	Lower trunk brachial plexopathy	No impairment
6	M/58	High	Clavicle Fx	1.5	Incomplete	AN, MCN	SSP, D, BB, FCU, EDC	Upper to middle trunk brachial plexopathy	Mild impairment
7	M/24	High	Acromion Fx, acromioclavicular joint injury	1	Complete	AN, MCN, SSN	SSP, D, BB	Upper trunk brachial plexopathy	Mild impairment
8	F/77	High	Acromion Fx	3	Incomplete	MN, UN	FCR, FDI, APB	Lower trunk brachial plexopathy	Mild impairment
9	M/84	High	Clavicle Fx	7	Incomplete	UN	FCU, FDI	UN	Mild impairment
10	M/42	High	Clavicle Fx	1	Incomplete	None	FCU, FDI, APB	Lower trunk brachial plexopathy	No impairment
11	M/35	High	Clavicle Fx	3	Incomplete	None	FDI, APB	Lower trunk brachial plexopathy	No impairment
12	F/37	High	Acromion Fx, coracoid Fx	2	Incomplete	UN	FDI, APB	Lower trunk brachial plexopathy	No impairment

EDX, electrodiagnosis; NCS, nerve conduction study; EMG, electromyography; Fx, fracture; AN, axillary nerve; MCN, musculocutaneous nerve; SSN, suprascapular nerve; RN, radial nerve; MN, median nerve; UN, ulnar nerve; SSP, supraspinatus; D, deltoid; BB, biceps brachii; TB, triceps brachii; FCR, flexor carpi radialis; FCU, flexor carpi ulnaris; EDC, extensor digitorum communis; APB, abductor pollicis brevis; FDI, first dorsal interosseous.

**Table 4:** Detailed Description of the Peripheral Nerve Injuries in Eight Patients with Other Causes of Shoulder Injury.

No	Sex/Age	Energy	Injury Area	Interval to EDX	Degree of Weakness at Onset	Motor NCS Abnormality	Denervated Muscles on Needle EMG	Affected Nerve	Recovery
1	F/65	High	RCT	2	Incomplete	AN, MCN	Multifidus, SSP, D, BB	C5-6 root	Mild improvement
2	M/70	High	RCT	1.5	Incomplete	AN	D	AN	Mild improvement
3	M/83	High	RCT	8	Complete	AN, MCN, SSN	SSP, D, BB	Upper trunk brachial plexus	Impairment
4	F/66	High	Scapular body Fx	6	Incomplete	AN, MCN, SSN	SSP, D, BB	Upper trunk brachial plexus	No impairment
5	M/53	High	Scapular body Fx	1	Complete	AN, RN	D, TB, EDC, FCR	Upper to middle trunk brachial plexus	Impairment
6	M/39	High	Scapular body Fx	9	Incomplete	AN, SSN	D, TB, FCU, EDC, FDI	Pan-brachial plexus	No impairment
7	M/52	High	Open wound	12	Incomplete	MCN	BB	MCN	Mild improvement
8	M/32	High	Stab injury at axilla	2	Complete	MN	FCR, FPL, APB	MN	Impairment

EDX, electrodiagnosis; NCS, nerve conduction study; EMG, electromyography; RCT, rotator cuff tear; Fx, fracture; AN, axillary nerve; MCN, musculocutaneous nerve; SSN, suprascapular nerve; RN, radial nerve; MN, median nerve; SSP, supraspinatus; D, deltoid; BB, biceps brachii; TB, triceps brachii; FCR, flexor carpi radialis; FCU, flexor carpi ulnaris; APB, abductor pollicis brevis; FDI, first dorsal interosseous; PQ, pronator quadratus; FPL, flexor pollicis longus.

**Table 5:** Summary of Peripheral Nerve Injuries Based on the Electrodiagnostic Investigation in Patients with Shoulder Trauma.

Pattern	Number	Energy	Recovery
Isolated AN	18	High 10 Low 8	Impairment 2 Mild impairment 9 No impairment 7
AN + MCN	6	High 2 Low 4	Impairment 2 Mild impairment 3 No impairment 1
Pan-brachial plexus	6	High 5 Low 1	Impairment 4 No impairment 2
Mainly upper trunk	5	High 5	Impairment 2 Mild impairment 2 No impairment 1
Mainly lower trunk	5	High 5	Mild impairment 1 No impairment 4
Isolated UN	4	High 3 Low 1	Impairment 1 Mild impairment 2 No impairment 1
Isolated MN	3	High 1 Low 2	Impairment 2 No impairment 1
Isolated RN	2	High 2	No impairment 2
C5-6 root	2	High 2	Mild impairment 2
Isolated MCN	1	High 1	Mild impairment 1
AN + SSN	1	Low 1	Mild impairment 1
AN + MN	1	High 1	Impairment 1
AN + UN	1	High 1	Mild impairment 1
AN + C5-6	1	High 1	No impairment 1

AN, axillary nerve; MCN, musculocutaneous nerve; SSN, suprascapular nerve; RN, radial nerve; MN, median nerve; UN, ulnar nerve.

**Table 6:** Articles Reporting Peripheral Nerve Injuries Following Shoulder Trauma.

Article	Number of Patients with Nerve Injury	Mean Age	Trauma Mechanism (% or Number)	Nerve Injury Distribution (% or Number)
Robinson et al. [4]	492 patients with dislocation (out of 3633 patients) (13.5%)	47.6	Fall 68.1% Sports accident 22.2% Motor vehicle accident 5.7% Others 4.1%	AN 66.9% UN 10.6% RN 1.8% MCN 1.2% MN 2.8% Multiple 16.7%
deLaat et al. [6]	101 patients (44 dislocation, 57 humeral neck fracture) 14 nerve lesions	63.5	Fall 87 Sports accident 14	AN 37 SSN 29 RN 22 MCN 19 UN 8 (duplication allowed)
Palisa et al. [14]	44 patients with dislocation (out of 226)	Most >50 years	Not available	AN 19 Multiple 25
Visser et al. [8]	37 patients with dislocation (out of 77)	52.3	Not available	AN 32 RN 5 MCN 9 UN 6 MN 3 (duplication allowed)
Visser et al. [9]	133 patients (37 dislocation, 96 proximal humerus fracture)	64.2	Not available	AN 53% SSN 37% RN 24% MCN 4% UN 12% MN 7% (duplication allowed)
Payne et al. [15]	48 patients (shoulder trauma)	45.5	Fall 17 Motor vehicle accident 14 Postsurgical accident 9 Lifting 7 Traumatic repetitive movement 2	Brachial plexopathy 20 AN 8 SSN 4 LTN 3 MCN 1 MACN 1 Multiple 11 (AN + MCN 5, AN + SSN 4, other 2)
Hems and Mahmood [10]	101 patients (dislocation 55, dislocation-like 20, proximal humerus fracture 15, hyperextension 11)	52	Fall 55 Motor vehicle accident 24 Hyperextension 11 Sports accident 6 others 5	AN 72 SSN 21 MCN 40 RN 54 MN 51 UN 56 C5/6 or upper trunk 3(duplication allowed)
Gutkowska et al. [13]	73 patients with dislocation	50	Fall 58 Motor vehicle accident 7 others 8	AN 54 MCN 21 RN 45 MN 48 UN 51 (duplication allowed)

AN, axillary nerve; MCN, musculocutaneous nerve; SSN, suprascapular nerve; LTN, long thoracic nerve; RN, radial nerve; MN, median nerve; UN, ulnar nerve; MACN. Medial antebrachial cutaneous nerve.
